# Are All Surgical Referrals for Endoscopic Retrograde Cholangiopancreatography Necessary?

**DOI:** 10.4103/1319-3767.45068

**Published:** 2009-01

**Authors:** Marco E. Malahias, Ebtisam Bsis

**Affiliations:** General Surgical Department, Dartford and Gravesham NHS Trust, Darent Valley Hospital, Dartford, Kent, DA2 8DA, UK. E-mail: thanner@emea.att.com

Sir,

Endoscopic retrograde cholangiopancreatography (ERCP) is the gold standard for the diagnosis of biliary obstruction.[1]

The objective of this study was to evaluate the necessity of emergency ERCPs in the acute phase (first 72 h) regardless of the trend of the liver function tests, thus exposing acutely ill patients to the risks of an invasive procedure[2] in the post magnetic resonance cholangiopancreatography (MRCP) era.[3,4]

There is no doubt that ERCP is a valuable and useful tool in management of hepatobiliary conditions. With less invasive diagnostic modalities, such as MRCP being available, the dogma of ERCP as a “gold standard” had to be re-evaluated.

Between December 2003 and December 2006, 73 consecutive ERCPs requested by surgeons in our unit, in a district general hospital of the United Kingdom, were retrospectively analyzed.

Indication and findings were then correlated with the trend in liver function tests:

73 patients (27 male and 46 female; median age, 57.95; and age range, 20–101 years) underwent ERCP during that three-year period.

The indications at time of presentation were pancreatitis in 10 patients (13.7%, group I) and jaundice in 63 patients (86.3%, group II).

Liver function test profile returned back to normal in 37 patients (50.7%, all from group II), improved in 10 patients (13.7%, three from group I, seven from group II), remained stagnant in 10 patients (13.7%, four from group I, six from group II), and deteriorated in 16 (21.9%, two from group I,14 from group II) before the emergency ERCP.

ERCP findings did not reveal any stones in the first group; however, it demonstrated bile duct stones in all patients in the second group, requiring stone extraction and sphincterotomy.

Our study shows that a percentage of our patients did indeed need ERCP; all of group II had calculi that required removal.

It was however only 28% of patients (26 of 73) that had stagnant or deteriorating liver function tests, thus warranting an emergency ERCP in the acute setting (first 72 h), while the patients were acutely unwell.

The outcome and conclusion of this study is that a supervised delay, with stringent monitoring of the trend of liver markers, will give many patients time to recuperate from their acute illness before an invasive procedure. This will also allow less invasive procedures, e.g., MRCP, play a role in selecting candidates for more invasive forms of management.
